# Supplementary material for: Temperature Responsive Diblock Polymer Brushes as Nanoreactors for Silver Nanoparticles Catalysis
Source: Polymers (Basel). 2023 Apr 19;15(8):1932. doi: 10.3390/polym15081932 (PMC10146612; doi:10.3390/polym15081932)
Supplement: Supplementary file 1 [file polymers-15-01932-s001.zip › polymers-2339991-supplementary.pdf]

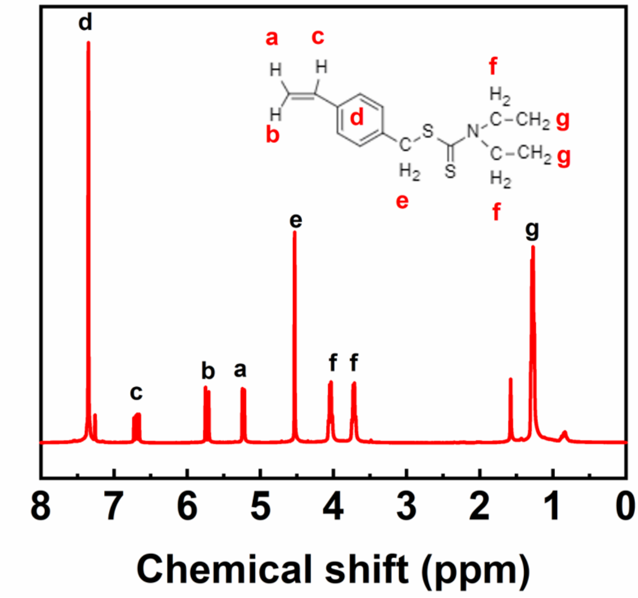

Figure S1. ^{1}H NMR spectra of VBDC.

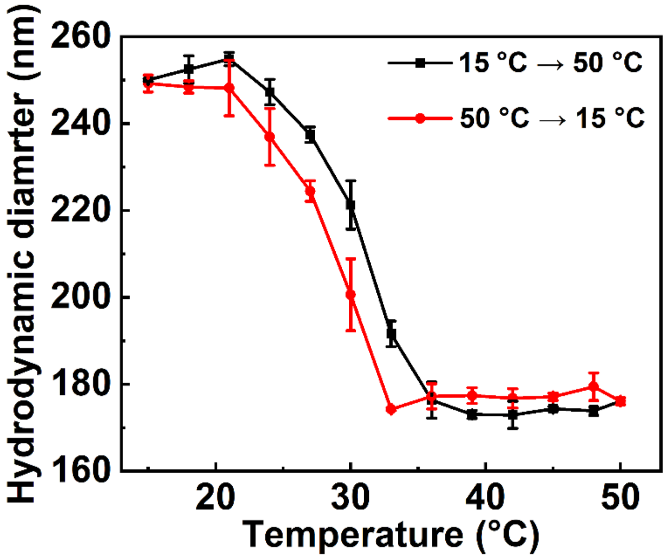

Figure S2. The diameter of PSV@PNIPA with temperature.

Table S1. The specific amount of substances in the reduction of 4-NP to 4-AP.

	**1**	**2**
PSV@PSS-b-PNIPA@Ag (μL)	43	
PSV@PNIPA-b-PSS@Ag (μL)		46
20 mM $NaBH_4$ (mL)	1.5	1.5
0.2 mM 4-NP (mL)	1.5	1.5

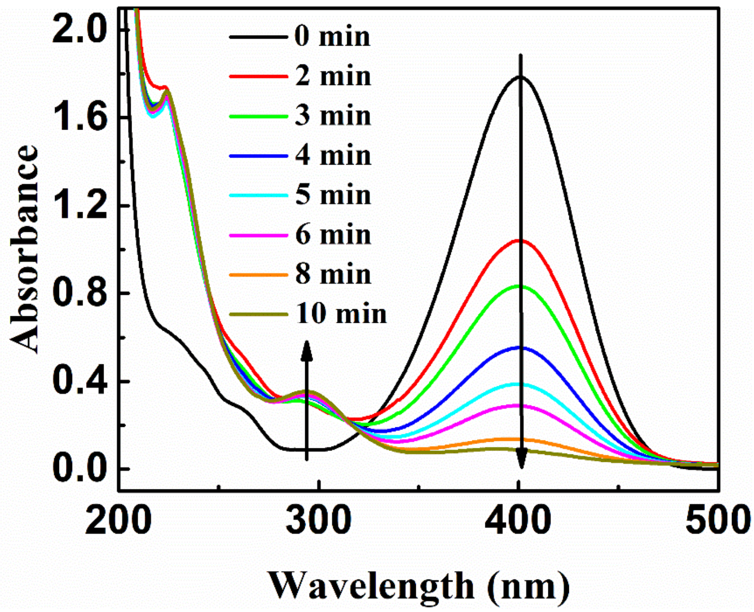

Figure S3. UV adsorption spectra of 4-NP to 4-AP catalyzed by diblock polymer brushes loading AgNPs.
